# Seebeck coefficient of silicon nanowire forests doped by thermal diffusion

**DOI:** 10.3762/bjnano.11.153

**Published:** 2020-11-11

**Authors:** Shaimaa Elyamny, Elisabetta Dimaggio, Giovanni Pennelli

**Affiliations:** 1 Dipartimento di Ingegneria della Informazione, Università di Pisa, Via G.Caruso, I-56122 Pisa, Italy; 2 Electronic Materials Research Department, Advanced Technology and New Materials Research Institute, City of Scientific Research and Technological Applications (SRTA-City), New Borg El-Arab City, 21934, Alexandria, Egypt

**Keywords:** nanowires, Seebeck coefficient, thermal conductivity, thermoelectricity

## Abstract

Thermoelectric generators made by large arrays of nanowires perpendicular to a silicon substrate, that is, so-called silicon nanowire forests are fabricated on large areas by an inexpensive metal-assisted etching technique. After fabrication, a thermal diffusion process is used for doping the nanowire forest with phosphorous. A suitable experimental technique has been developed for the measurement of the Seebeck coefficient under static conditions, and results are reported for different doping parameters. These results are in good agreement with numerical simulations of the doping process applied to silicon nanowires. These devices, based on doped nanowire forests, offer a possible route for the exploitation of the high power factor of silicon, which, combined with the very low thermal conductivity of nanostructures, will yield a high efficiency of the conversion of thermal to electrical energy.

## Introduction

Thermoelectric generators for direct conversion of heat into electrical power will certainly play a decisive role in the next generation of energy harvesting and energy scavenging systems. However, a large-scale application of thermoelectric devices requires the development of materials that have good thermoelectric features and are, at the same time, of low cost, technologically affordable and sustainable. Silicon has a very high power factor *S*^2^σ [1–4] (*S* is the Seebeck coefficient and σ is the electrical conductivity). This, combined with the reduced thermal conductivity when nanostructured [5–10], makes it very suitable for thermoelectric applications. As added value, silicon is cheap and abundant. Also, there are established technologies for processing Si and Si is biocompatible. Furthermore, the use of silicon for thermoelectric generator devices will make them technologically compatible with standard CMOS devices. The main requirement for the use of silicon as thermoelectric material is the development of techniques for the low-cost fabrication and interconnection of a large number of nanostructures to generate a significant amount of power. Metal-assisted chemical etching (MACE) [11–14] of silicon is very promising because it gives the opportunity to fabricate large numbers of nanowires with high aspect ratio, perpendicular to a silicon substrate, that is, so-called silicon nanowire (SiNW) forests. The process is very suitable for the large-scale fabrication of nanostructured devices useful for several applications, such as sensing, photovoltaics, energy storage (supercapacitors), and, in particular, thermoelectric applications [15–17]. There are two main requirements to the fabrication of a leg for a thermoelectric generator that is based on a large number of silicon nanowires perpendicular to a substrate: 1) Electrical contacts need to fabricated on the top of a silicon nanowire forest, which can be achieved by copper electrodeposition [18]. 2) The optimum doping concentration of the nanowires for the exploitation of the maximum power factor of silicon [3] needs to be found. Both the Seebeck coefficient and the electrical conductivity depend on the doping concentration. In particular, *S* decreases with increasing doping concentrations. At the same time, high doping concentrations yield high values of electrical conductivity σ. Hence, a trade-off between *S* and σ needs to be found, such that the product *S*^2^σ is maximized. Nanowires with an average diameter of 80 nm and a length of several hundreds of micrometers can easily be obtained from MACE on n- and p-doped substrates, with concentrations up to 10^18^ cm^−3^. However, the maximum of the power factor is achieved for doping concentrations greater than 10^19^ cm^−3^ [2–3]. Unfortunately MACE yields very unreliable results at such high doping concentrations. In the case of p^+^ doping, parameters for a satisfactory fabrication of monocrystalline SiNW forests have been found [17]. However, the reliable fabrication of n^+^ SiNWs by MACE is still an open issue [19–20]. Hence, it is currently not possible to fabricate an optimized generator module based on two legs with opposite heavy doping.

A possible solution is to dope the silicon nanowires by thermal diffusion [21], after their fabrication by MACE. In this work, we present the measurement of the Seebeck coefficient of SiNWs doped through a diffusion process, based on a solid source (see Methods section). The main point is that the diffusion process must be performed in a single step (predeposition step), because a drive-in step, typical of the standard diffusion processes currently applied in the semiconductor industry, would require an oxidation of the surface for the trapping of the doping species. However, the reduction of the thermal conductivity, which is the aim of the nanoscale structuring, relies on the roughness of the nanowire surfaces, and the smoothing produced by the oxide growth would heavily reduce this effect. Therefore, it is mandatory to perform a single-step diffusion process, which results in a nonuniform doping concentration in the nanowire. Here, we report the measurement of the Seebeck coefficient after different doping processes, and compare the measurements with numerical simulations that take into consideration the nonuniform doping of the silicon nanowires. We found a very good agreement between experimental measurements and simulations of the doping process.

## Methods

### Fabrication and doping of silicon nanowire forests

**Figure 1:**
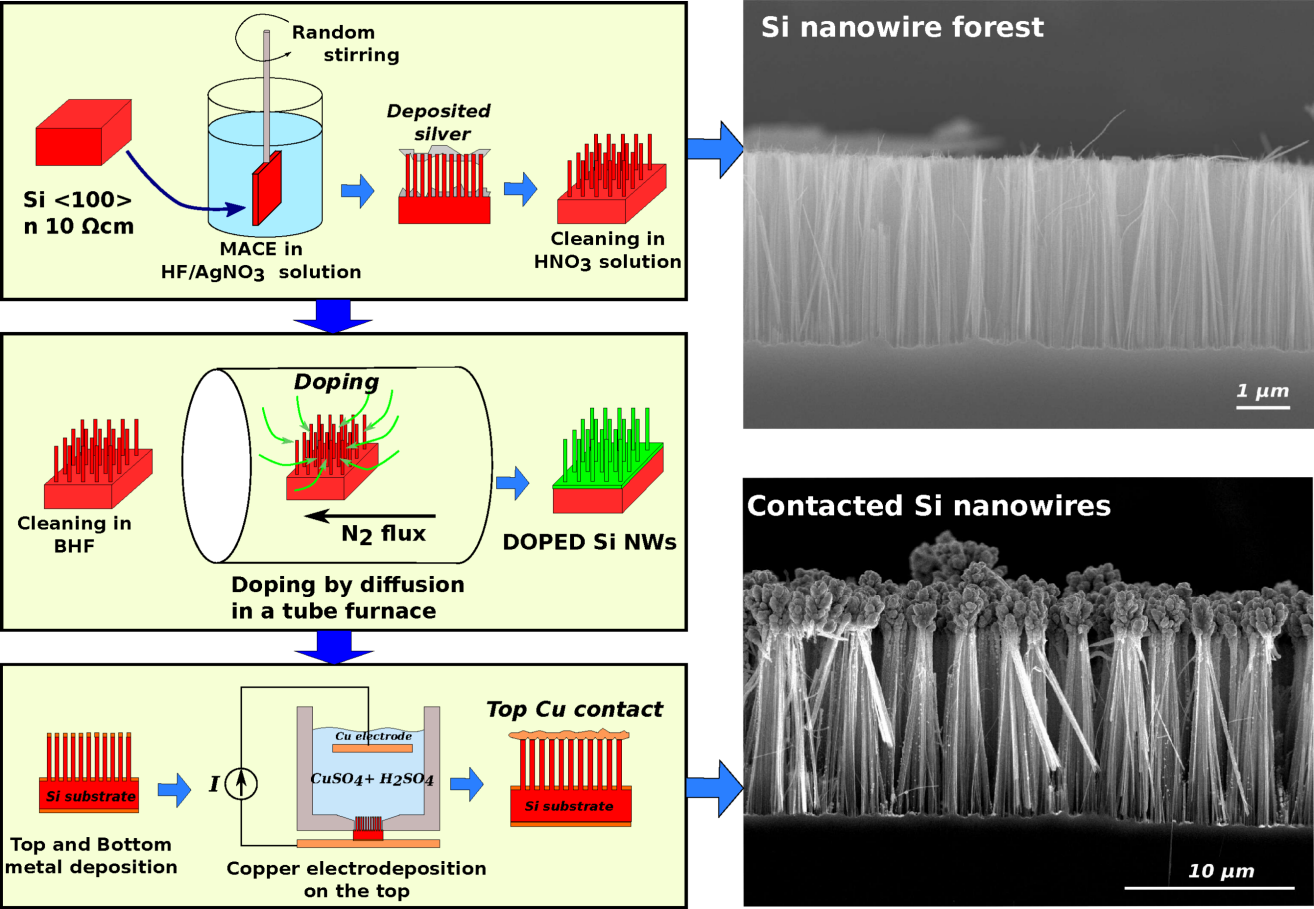
(Left) Schematics of the fabrication process and (right) SEM images of silicon nanowire forests. The forests consist of nanowires with a length of several tens of micrometers, placed perpendicularly to the silicon substrate.

Silicon nanowire forests have been fabricated by a simple and inexpensive process based on one-pot metal-assisted chemical etching (MACE) [22] (Figure 1).

Silicon chips of roughly 1 × 1 cm^2^ have been cut from n-doped (phosphorous) commercial silicon ⟨100⟩ wafers with a nominal resistivity of 10 Ω·cm (nominal doping concentration 10^15^ cm^−3^). The chips, mounted on a custom-made apparatus to provide the stirring during the etch, have been soaked in a HF/AgNO_3_ solution (HF (48%)/AgNO_3_ (0.1 M)/H_2_O, volume ratio 16:5:60, see Figure 1). The stirring is crucial for the uniformity of the etching. Hence, the custom-made apparatus that holds the sample changes at random (every few seconds) the direction and the speed of the rotation. During the etch, a temperature-controlled bath allowed to maintain a stable temperature of 18 ± 0.5 °C. The etching time determines the final length of the nanowires. Several SiNW forests with nanowire lengths between 6.5 μm (30 min etching) and 41 μm (3 h etching) have been fabricated for this work. At the end of the MACE process step, the chips were covered with granular silver, which has been removed by etching in a HNO_3_/H_2_O 1:1 solution for 2 min. SEM inspection confirmed the complete removal of silver from the SiNW forests, which are covered by a thin layer of oxide as a result of the HNO_3_ etching.

The doping of the silicon nanowires has been carried out by thermal diffusion from a solid source. At first, the chips with the SiNW forests, with a surface of roughly 1 × 1 cm^2^ have been cleaned in buffered HF (BHF) for 1 min, to remove the SiO_2_ grown during the HNO_3_ etching. The chips have then been placed in a tubular quartz furnace together with the solid source. As a solid source, we used ceramic wafers provided by Techneglass (PhosPlus TP-250). The face of the chips with the nanowires has been placed in contact with the ceramic wafer. The sealed tube has been cleaned with a flux of pure nitrogen for several minutes; the nitrogen flux has been maintained during the whole thermal doping process. The temperature has been raised to the target temperature, with a ramp of 20 °C/min. Once the target temperature had been reached, it has been maintained for the chosen doping time (ten minutes, typically). Then, the chips have been allowed to cool maintaining the nitrogen flux.

After doping by thermal diffusion, a contact has been provided on top of the silicon nanowire forest exploiting the copper electrodeposition method described in a previous publication [18]. At first, a Cr (for adhesion)/Cu double metal layer has been deposited by thermal evaporation both on top of the nanowires and on the other side of the silicon wafer, that is, at the bottom of the substrate. The Cu layer on the top has been used as a seed for the electrodeposition of copper, performed at a constant current of 800 A/m^2^ for 2 min.

At the end of the process, we obtained a SiNW forest placed between a top copper contact and the silicon substrate, contacted through the bottom metal layer. The doping of the SiNW forest depends on the diffusion process (temperature and time), while the substrate remains slightly doped, with a nominal resistivity of 10 Ω·cm. The result is a leg of a silicon-based thermoelectric generator, as shown in Figure 1.

### Measurement of the thermal conductivity and of the Seebeck coefficient

**Figure 2:**
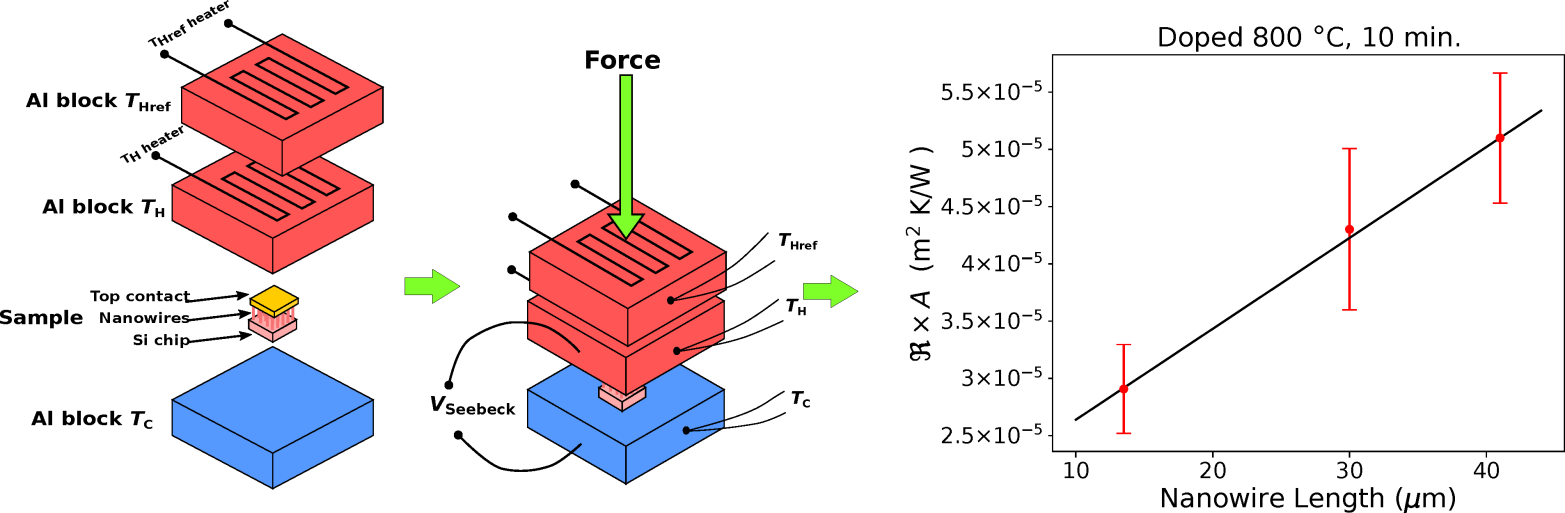
Left: sketch of the measurement apparatus. Right: thermal resistance of several samples as a function of the nanowire length (doping at 800 °C for 10 min).

Single-leg thermoelectric generators have been characterized with a measurement apparatus based on the guarded hot-plate technique, schematically shown in Figure 2.

This apparatus has already been used in a previous publication for the measurement of the thermal conductivity of undoped silicon nanowire forests [23]. Essentially, the Si chip with the contacted nanowire forest is squeezed between two aluminum blocks maintained at different temperatures *T*_H_ = *T*_Hot_ and *T*_C_ = *T*_Cold_. Given the surface *A* of the sample, the squeezing pressure has been set to 20 MPa for all measured samples. A third block *T*_HRef_ is maintained at the same temperature as *T*_H_ (within ±0.1°), so that the electrical power needed to heat *T*_H_ through Joule heating is the thermal power 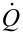 = *V* × *I* that crosses the sample in the absence of heat losses. The system is in vacuum and the irradiation loss is limited by a thermal shield maintained at *T*_H_. Hence, the thermal resistance ℜ can be evaluated as ℜ = (*T*_H_ − *T*_C_)/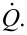 Finite-element modeling has been used to refine the temperature measurement (see our previous publication [23] for more details). Samples of different lengths, obtained after different etching times, and with different doping temperatures, have been fabricated and measured. The plot of Figure 2 shows ℜ × *A* for several samples, where *A* is the area of the sample, as a function of the nanowire length.

Each point is the average of multiple measurements on the same sample, achieved with different thermal power values, 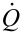 = *V* × *I*, between 0.5 and 2 W. Each measurement has been made under static conditions. The electrical power *V* × *I* has been applied to the *T*_H_ heater, and an automatic system controlled the heater of the *T*_HRef_ block, so that *T*_Href_ was within ±0.1° from *T*_H_. Once the electrical power *V* × *I* had been applied, a sufficiently long time (roughly 1 h) has been waited for the thermal stabilization of the system, before recording *T*_H_, *T*_C_ and the thermal power 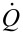 through the sample. The linear fit of ℜ × *A* as a function of the nanowire length *L* is also reported in the plot of Figure 2. The reciprocal of the slope is the thermal conductivity *k*_t_, multiplied by the coverage factor (or filling factor) ν, where ν is the ratio between the total cross-section surface of the nanowires and the surface of the sample. From the plot, a value of *k*_t_ = 4.2 ± 0.4 results for the samples doped at 800 °C.

For the estimation of the filling factor, several SEM images have been taken of different locations of each sample. The area of the samples is roughly 1 × 1 cm^2^, each image covers an surface of at least 50 × 50 μm^2^. A software for image reduction (ImageJ) has been used to determine the filling factor in each image, and an average has been calculated repeating the procedure for at least ten images. The resulting average filling factor was ν = 0.3 ± 0.02. These values are comparable, within the experimental errors, with that measured on undoped samples, reported in our previous work [8]. The intercept with the vertical axis of the linear fits, shown in the plots of Figure 2, is the thermal resistance of the contacts ℜ_C_*A* = (1.8 ± 0.6) × 10^−5^ m^2^·K/W. It has been assumed that the contact thermal resistance ℜ_C_*A* was the same for all the samples, since it depends on the mechanical pressure, which has been set to 20 MPa during all measurements.

The thermal resistance of the contacts ℜ_C_*A* is fundamental for the evaluation of the Seebeck coefficient *S*. Simultaneously to the measurement of the thermal resistance of each sample, the voltage between the aluminum blocks *T*_H_ and *T*_C_ has been measured (Seebeck voltage *V*_seebeck_ for *T*_H_ − *T*_C_). However, the temperature difference Δ*T* = *T*_H_ − *T*_C_ includes also the temperature drop due to the contact thermal resistance, that is, Δ*T* = Δ*T*_NW_ + Δ*T*_CONTACTS_. Hence, the evaluation of *S* simply as the ratio between the measured values of *V*_S_ and Δ*T* (*S* = *V*_S_/Δ*T*) would give an underestimated value, because Δ*T*_NW_ is smaller than the measured value of Δ*T* = *T*_H_ − *T*_C_. The measured value of the contact thermal resistance ℜ_C_*A*, together with the value of the heat flux through the sample 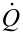 = *V* × *I*, can be used for a correct evaluation of Δ*T*_NW_:

[1]
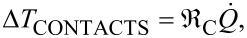


[2]
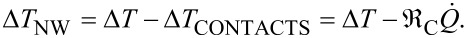


For each sample, several Seebeck voltage values *V*_seebeck_ have been recorded for different heating power values 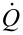, that is, for different values of Δ*T*. As explained in the description of the thermal resistance, each measurement was carried out under static conditions, allowing for a thermal stabilization of the system before recording the data. The temperature drop across the nanowires, Δ*T*_NW_, has then been evaluated exploiting the value of the thermal contact resistance.

## Results and Discussion

**Figure 3:**
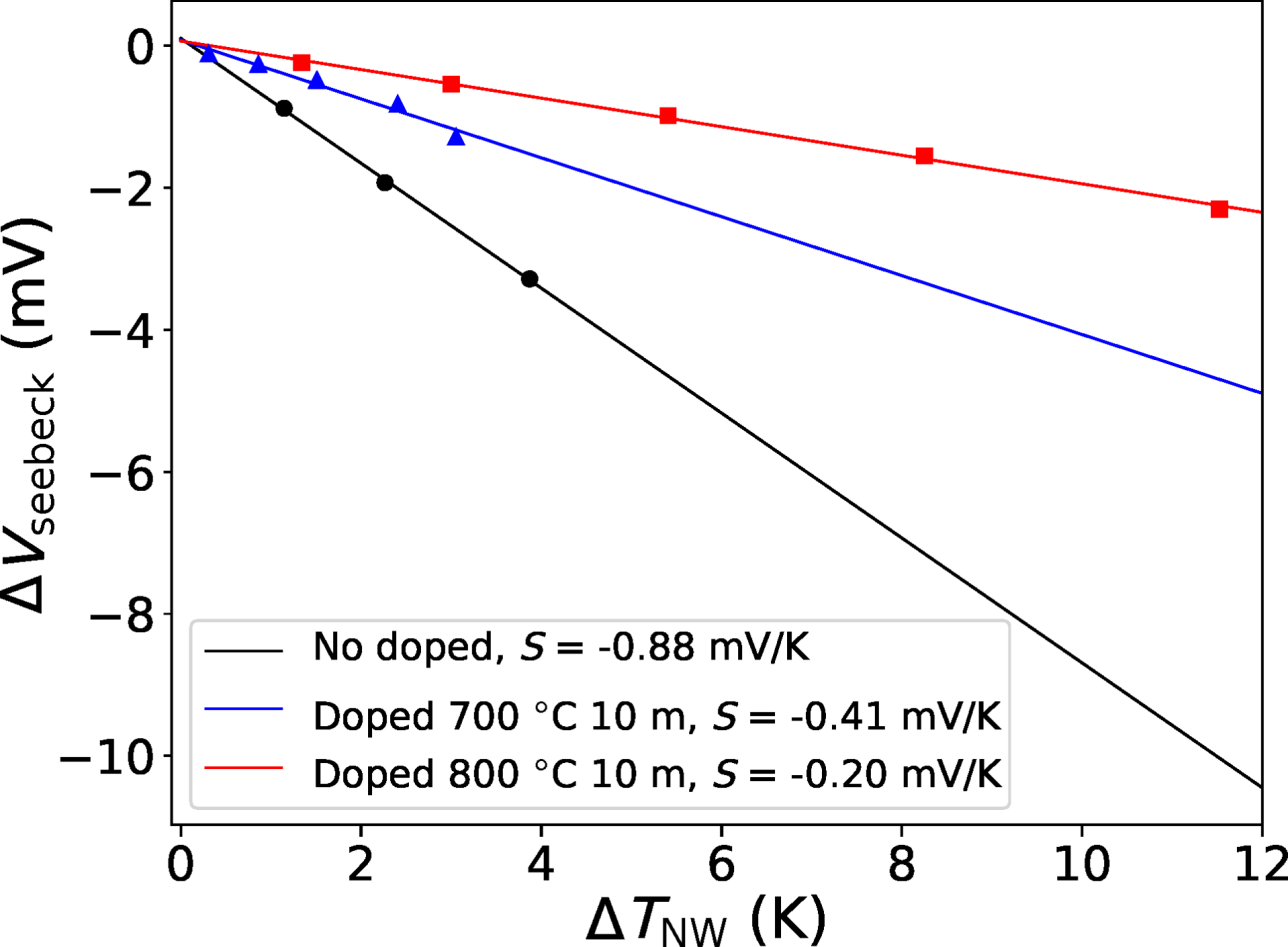
Measured voltage drop (Seebeck voltage Δ*V*_seebeck_) as a function of the temperature difference between the ends of the nanowires, Δ*T*_NW_. The slope is the Seebeck coefficient of the nanowires.

Figure 3 shows the measured voltage drop as a function of the temperature difference between the ends of the nanowires, calculated as described above. The slope of the linear fit gives the Seebeck coefficient, reported in the legend of the figure for the different values of the doping process parameters (doping temperature and time). The results are *S* = −0.88 mV/K for the undoped sample (nominal resistivity 10 Ω·cm, estimated doping concentration 10^15^ cm^−3^), *S* = −0.41 mV/K for the sample doped at 700 °C for 10 min, and *S* = −0.20 mV/K for the sample doped at 800 °C for 10 min.

In the case of undoped samples, it is very difficult to establish the final charge carrier concentration, because it is strongly affected by the surface states of the nanostructures. In the case of doped samples, it is presumable that the doping concentration inside the nanowires is not uniform, because phosporous diffuses from the surfaces generating an exponential (error function-like) profile inside the nanowire. It must be noted that the standard diffusion doping process, used in the fabrication of integrated devices, consists of two steps. First, there is a predeposition step, in presence of a phosphorous-rich atmosphere, which results in an exponential doping profile (such as the doping performed here). Second, there is a so-called drive-in process in oxidizing atmosphere. This second step, performed at temperatures in excess of 1000 °C, is necessary to allow for the penetration of the doping species (phosphorous, in our case) into the silicon. It requires an oxidizing environment, at least in a first preliminary phase, to grow a thin SiO_2_ layer at the surface as a barrier for the doping species, forcing the diffusion into silicon. In the specific case of thermoelectric applications, we cannot afford an oxidization process, because it would smooth the surfaces. The reduction of the thermal conductivity, which is essential for good thermoelectric features, relies on the roughness of the surfaces, which would be compromised by an oxidation step. Hence, the best solution is to perform the doping using only the predeposition step.

Given the doping parameters, we gave an estimation of the doping distribution into the nanowires by simulating the diffusion process. Phosphorous diffusion in silicon is a well-known process, widely characterized for its importance in the fabrication of integrated electronic devices and circuits [24].

**Figure 4:**
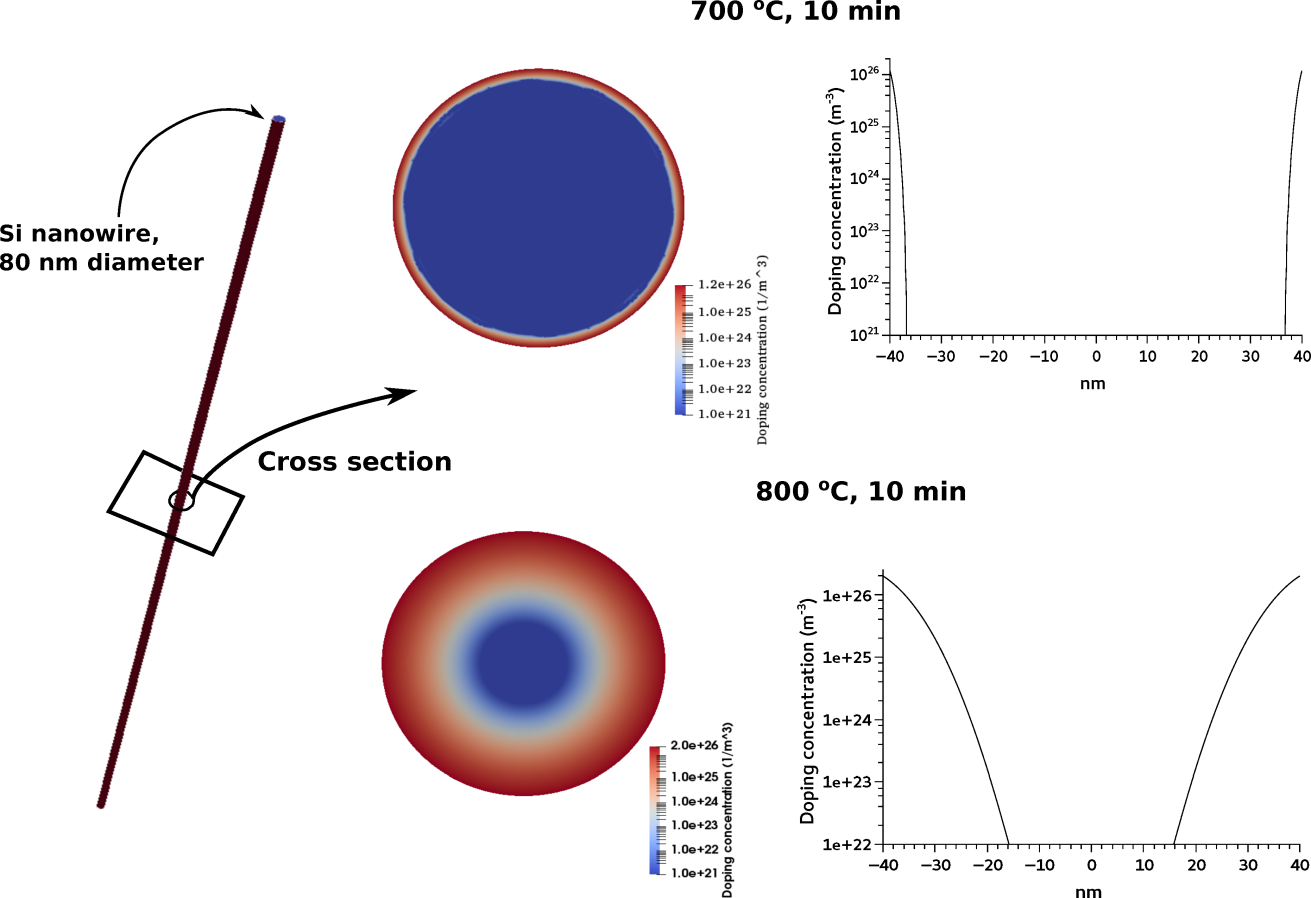
Numerical simulations of phosphorous diffusion in silicon nanowires. The doping concentration is maximum on the surfaces and decreases exponentially along the radius toward the center.

The diameter of the nanowires fabricated by MACE is not uniform, but it is distributed around an average value of 80 nm, which depends on the etching parameters. The transport properties (thermal conductivity and Seebeck coefficient) have been measured on macroscopic samples (several square millimeters of surface). Therefore, their value is averaged over a large number of nanowires with different diameters. For the simulations of the doping diffusion process, we considered the average diameter. Figure 4 shows finite-element simulations of doping diffusion in nanowires with a diameter of 80 nm and length of several micrometers.

The simulations were carried out solving the 2D diffusion equation 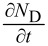 = −*D*_P_∇*N*_D_(*x*,*y*,*t*) in the circular cross section of a typical nanowire, where *N*_D_(*x*,*y*,*t*) is the doping concentration as a function of the position and of the time, *t* is the time and *D*_P_ is the diffusion coefficient of phosphorous at the given temperature *T*, evaluated as 
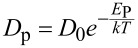
, with *D*_0_ = 0.79 cm^2^/s and *E*_P_ = 3.29 eV, following [24]. The radial doping profiles are also reported in Figure 4. The Seebeck coefficient of the doped nanowires is difficult to evaluate because the doping is not uniform. There are experimental papers [2,25–27] reporting the Seebeck coefficient *S* = *S*(*n*) measured on bulk silicon with uniform doping concentration *n*. As shown in our previous work [3], the best logarithmic fit of the reported experimental data is *S*(*n*) = 0.0077 – 1.26 × 10^−4^ ln(*n*) (*n* is the doping concentration in m^−3^, *S* as absolute value). This relationship between *S* = *S*(*n*) and the doping concentration can be used to evaluate the Seebeck coefficient in our nanowires with nonuniform doping. The doping profile has been assumed as *n*(*x*,*y*) = *N*_D_(*x*,*y*), where *N*_D_(*x*,*y*) is the result of the simulation of the doping process. Hence, the Seebeck coefficient depends on the position in the nanowire. That is, *S* = *S*(*n*(*x*,*y*)), where (*x*,*y*) is a generic point in the cross section. Also the electrical conductivity depends on doping, that is, σ(*x*,*y*) = σ(*n*(*x*,*y*)) It has been estimated using the formula by Arora [28], which is widely used in silicon device simulations. The Seebeck coefficient of the nanowires has then been calculated as:

[3]
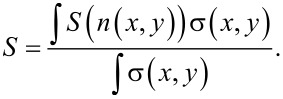


This formula can be easily derived by considering the nanowires as many parallel thermoelectric generators *S_i_*Δ*T*, each with its resistance *R_i_* (conductance *G_i_* = 1/*R_i_*). It is straightforward to obtain 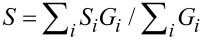. Taking into account the distributed parameters we derive the formula above. The simulations yield (as absolute values) *S* = 0.448 mV/K for the nanowires doped at 700 °C for 10 min, and *S* = 0.207 mV/K for the nanowires doped at 800 °C for 10 min. This values are in very good agreement with the experimental results.

## Conclusion

The development of efficient silicon-based thermoelectric generators require the improvement of low-cost processes for the fabrication of large assemblies of nanostructures, which also require an optimized doping concentration to achieve the maximum power factor. Metal-assisted chemical etching is a very advantageous technique, because it allows for the fabrication of silicon nanowires with high aspect ratio on a very large scale. However, MACE is incompatible with the high doping concentrations necessary for the optimization of the power factor. Our work is devoted to the investigation of the Seebeck coefficient of large-area silicon nanowire forests, doped by thermal diffusion after their fabrication. At first, we presented a reliable measurement procedure for the measurement of the Seebeck coefficient of macroscopic samples, made of large collections of nanometric structures (vertical silicon nanowires). Then, we demonstrated that the measured Seebeck coefficient is compatible with simulations of the diffusion process, which results in a nonuniform radial doping concentration.

Hence, the fabrication by MACE of slightly doped SiNW forests, and their doping by diffusion after fabrication, is a possible route for the exploitation of nanostructured silicon for thermoelectric purposes. Procedures for the fabrication of macroscopic nanostructured-Si generators, based on interconnected p- and n-doped legs, are under development.
